# Ex situ conservation of *Cymbidium eburneum* Lindl.: a threatened and vulnerable orchid, by asymbiotic seed germination

**DOI:** 10.1007/s13205-012-0062-8

**Published:** 2012-04-17

**Authors:** Kiran Gogoi, Suman Kumaria, Pramod Tandon

**Affiliations:** Plant Biotechnology Laboratory, Department of Botany, North-Eastern Hill University, Shillong, 793 022 India

**Keywords:** Ex situ conservation, Endangered, Asymbiotic seed germination, Protocorms, *Cymbidium eburneum*

## Abstract

The population of many splendid orchids is reducing from their natural habitats at an alarming rate and their conservation is becoming a matter of global concern. Asymbiotic seed germination has been applied for ex situ conservation of rare, endangered and threatened orchid taxa and could provide rapid means their multiplication. In the present study reported here, seeds of an epiphytic and rare orchid, *Cymbidium eburneum* were germinated asymbiotically in different basal media viz., Murashige and Skoog (MS), Knudson C, Mitra et al. (Mitra), Gamborg et al. (B_5_) and Nitsch. The highest germination rate was observed in Mitra medium, whereas the development of the protocorms was found to be best in MS medium. Effects of growth regulators viz., indole-3 acetic acid (IAA), α-naphthalene acetic acid (NAA), 2,4-dichlorophenoxyacetic acid (2,4-d), thidiazuron (TDZ), 6-benzyl aminopurine (BAP) and kinetin (Kn) both singly and in combination incorporated in the MS medium were studied on growth and development of seedlings. It was observed that MS medium nourished with 15 μM each of BAP and NAA in combination was found to enhance shoot number and length, and root number and length in the seedlings. The rooted seedlings were successfully acclimatized**.**

## Introduction

Orchids are members of the family *Orchidaceae*, which is the largest and the most specialized family of flowering plant. All the species of family *Orchidaceae* are listed in the Endangered Species of Wild Fauna and Flora in Appendix II of the Convention of International Trade (CITES, Chugh et al. 2009). Approximately 50 % of India’s orchid wealth, comprising 750–800 species, is found mainly in Northeastern India. Unfortunately, the orchid diversity in Northeast India and the country as a whole has been threatened due to increased biotic influences, socioeconomic development and uncontrolled commercial exploitation of forests.

**Fig. 1:**
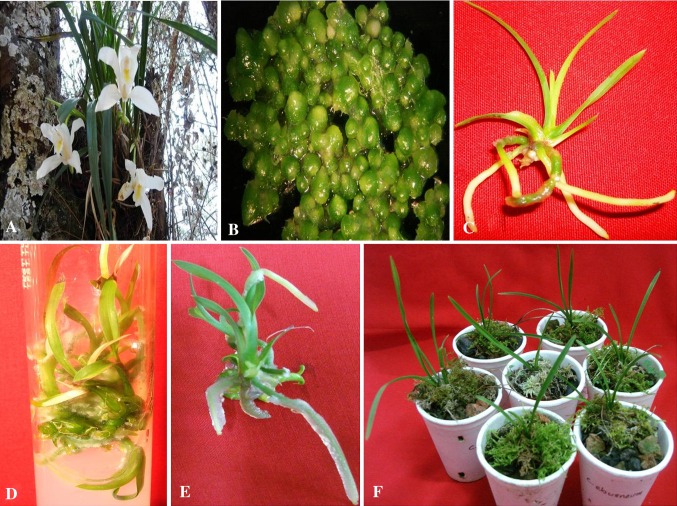
Ex situ conservation of *Cymbidium eburneum* by asymbiotic seed germination. **a***C. eburneum* blooming in its natural habitat, **b** asymbiotic seed germination in Mitra medium, **c** protocorm development in plantlets in MS medium, **d**, **e** multiple shoots and roots in MS medium supplemented with 15 μM each of BAP and NAA and **f** greenhouse acclimatized plantlets

The members of the genus *Cymbidium* are the most important commercial orchids primarily grown for flowers. *Cymbidium eburneum* Lindl. is endemic to Eastern Himalayas and Northeastern India, growing at elevations of 1,000–1,500 m above sea level. The flowers of *C. eburneum* bloom from winter to spring on an erect basally sheathed long inflorescence (Fig. 1a). This species has been widely used to confer large size and white color to its hybrids. *C. eburneum* is a threatened species and has been listed as vulnerable in the Red Data Book Plants of India, (Nayar and Sastry 1987–1988, 1999). Consequently, there is a need to protect and conserve this species. Less than 5 % of orchid seeds germinate in nature because the seeds are non-endospermous and require specific mycorrhizal fungi in the initial stages of development (Arditti and Ernst 1984; Kumaria and Tandon 2001). For ex situ conservation and reintroduction of endangered orchids, use of in vitro protocols is a successful approach (Arditti and Ernst 1984; Kumaria and Tandon 2001). Ex situ conservation with in vitro seed germination is more advantageous as the process increases adaptive evolutionary changes and as a result there is more genetic variation in reintroduced populations (Tandon and Kumaria 2005).The aim of this study was to develop a suitable strategy for ex situ conservation of *C. eburneum* through large-scale in vitro propagation.

## Materials and methods

### Capsule preparation and seed treatment

Nine-month-old hand pollinated green capsules of *C. eburneum* were collected from greenhouse-grown plants at Plant Biotechnology Laboratory, Department of Botany, North-Eastern Hill University, Shillong. The capsules were washed under running tap water, then in 10 % Teepol (Qualigens Fine Chemicals, Mumbai, India), surface sterilized using 0.04 % (w/v) Bavistin solution (BASF, Mumbai, India) for 20 min and washed three times with sterile double distilled water. The capsules were finally dipped in 70 % ethanol for 30 s followed by flaming for 2–3 s. The sterilized capsules were split longitudinally with a sterile surgical blade and the seeds were inoculated on the surface of agar amended nutrient media.

### Optimization of different culture media for seed germination

Five different media which included MS (Murashige and Skoog 1962), Nitsch (Nitsch 1969), B_5_ (Gamborg et al. 1968), Mitra (Mitra et al. 1976) and Knudson C (Knudson 1946) were tested to select the best basal medium for maximum seed germination. The media were supplemented with 3 % sucrose and solidified with 0.8 % agar (HiMedia Laboratories, Mumbai, India). The pH of the media was adjusted to pH 5.8 prior to sterilization by autoclaving for all the above experiments. Culture tubes were incubated at 25 ± 2 °C under 12 h photoperiod of 60 μmol^−2^ s^−1^ light intensity.

The percentage germination of seeds in different media was determined by the formation of protocorms. Observations of different parameters such as the time required for protocorm formation, color of protocorm and percentage (%) of seed germination, shoot number and length, and root number and length were recorded after 8 weeks of seed inoculation. The seeds were randomly removed and dispersed in a drop of water on a glass slide and observed under a light microscope. The swollen seeds (the spherules were formed) were regarded as germinated and unswollen seeds as ungerminated. The seed germination percentage was calculated by the following formula (Hossain et al. 2010; Roy et al. 2011): % seed germination = (number of seeds forming spherule/total number of seeds) × 100.

### Optimization of culture media on growth and development of protocorms and seedlings

Protocorms of *C. eburneum* were cultured in five different media: MS, Mitra, Nitsch, B_5_ and Knudson C described above. The medium on which seedlings developed best was further considered for studying the effect of auxins. For this, 3-indole acetic acid (IAA), α-naphthaleneacetic acid (NAA) and 2,4-dichlorophenoxyacetic acid (2,4-d), and the cytokinins viz., 6-benzyle amino purine (BAP), thidiazuron (TDZ) and kinetin (Kn) at different concentrations (5, 10, 15, 20 μM) both singly and in various combinations were added to the best medium and ten seedlings inoculated. Observations of seedlings were performed after 4 weeks of culture and different growth parameters viz., shoot number and length, and root number and length were recorded.

### Acclimatization and transfer of in vitro-raised plantlets to soil

Complete in vitro regenerated plantlets of *C. eburneum* measuring about 2.5–3.0 cm in height were transferred to thermocol pots containing different compost mixtures of brick, charcoal and decaying litter in the different ratios viz., (1) brick and charcoal pieces (1:1), (2) brick and decaying litter (1:1), (3) brick pieces, charcoal pieces and decaying litter (1:1:1), and (4) brick pieces, charcoal pieces and decaying litter (1:1:1) + layer of moss. The temperature of the glass house for acclimatization was maintained at 25 ± 2 °C. The relative humidity of the glass house was around 70–80 % and the plantlets were watered daily. Readings were recorded after 12 weeks of transfer.

### Statistical analysis

The experiments were repeated three times with ten replicates per treatment. Statistical analysis was done by analysis of variance (ANOVA) at *p* < 0.05 and means compared using Tukey’s test (PC version Origin 7.0 NORTHAMPTON, MA, USA).

## Results and discussion

### Asymbiotic seed germination and early seedling development

In the present study, it was observed that, of the different media for asymbiotic seed germination of *C. eburneum*, the highest seed germination of 98.3 % was recorded on Mitra basal medium after 8 weeks of inoculation followed by MS (87.4 %), Knudson C (77.3 %), B_5_ (64.2 %) and the lowest in Nitsch (53 %) (Table 1; Fig. 1b, c). The protocorm formation was observed earliest in Mitra medium after 8 weeks followed by MS and KC (9 weeks), whereas in B_5_ and Nitsch media maximum time was required for the formation of protocorms (11 and 12 weeks, respectively). In Nitsch medium, the protocorms were whitish and turned brown eventually. The high seed germination of *C. eburneum* in Mitra medium might have been influenced by the presence of major ions in reduced amounts with chlorine being absent as compared to Knudson C, B_5_, MS or Nitsch media*.* This finding is similar to that reported by Hossain et al. (2010) in *Cymbidium giganteum*. Also, the additional presence of riboflavin, biotin and folic acid in Mitra medium might have further enhanced seed germination. Mitra medium has higher concentration of phosphate ion (PO_4_^3−^) compared to the other media tried. Effects of phosphate on asymbiotic orchid seed germination of other orchids have also been reported (Dutra et al. 2008). It has also been shown by Dutra et al*.* (2008) that nitrogen does not play an important role in asymbiotic orchid seed germination as other nutrients. Similarly, in this study, it was found that the low total nitrogen content in Mitra medium (3.39 g) as compared to MS (60.05 g) and B_5_ (25.76 g) media did not affect seed germination of *C. eburneum*. Hajong et al. (2010) reported that the low response of orchid seeds in B_5_ and Nitsch media might be due to the inhibitory influence of nitrogen in the form of ammonium sulfate on seedling growth in B_5_ medium or mixtures of vitamins present in both B_5_ and Nitsch media.

**Table 1:** Effect of different media on seed germination, growth and development of *Cymbidium eburneum*

Media	Germination (%)^#^	Time taken for protocorm formation (in weeks)	Color of protocorms	Average shoot number^#^	Average shoot length (cm)^#^	Average root number^#^	Average root length (cm)^#^
MS	87.4 ± 2.1^cd^	9	Light green	2.2 ± 0.4^b^	3.8 ± 0.1^c^	3.8 ± 0.4^b^	3.7 ± 0.2^b^
B5	64.2 ± 2.1^b^	11	Light green	1.4 ± 0.2^b^	2.5 ± 0.1^b^	0	0
Mitra	98.3 ± 1.1^d^	8	Dark green	1.6 ± 0.2^b^	3.1 ± 0.2^bc^	2.4 ± 0.2^a^	2.8 ± 0.2^a^
Knudson C	77.3 ± 1.5^bc^	9	Light green to whitish	0.4 ± 0.2^a^	0.6 ± 0.4^a^	0	0
Nitsch	53.0 ± 1.5^a^	12	Whitish	0.2 ± 0.2^a^	0.3 ± 0.2^a^	0	0

Values are mean ± SEM of three experiments with ten replicates/experiment

ANOVA test shows that seed germination is highly significant at 5 % level

Means followed by the same letter are not significantly different according to Tukey’s test (*p* = 0.05)

^#^Data shown are the mean of ten replicates ± standard error (SE)

The nutrient requirement of orchid seeds varies at different stages of development. In our study, we found that protocorm development into plantlets with highest number of shoots (2.2) and roots (3.7) was observed in MS medium followed by Mitra medium (1.6 average number of shoots and 2.4 roots). However, in both KC and Nitsch, the average number of shoots (0.4 and 0.2, respectively) was poor and rooting did not take place in the shoots (Table 1). Protocorm development into seedlings was more pronounced in MS medium and this might be attributed to the availability of micro and macronutrients, vitamins, inositol and glycine in high amounts as compared to other media. High concentration of nitrogen, i.e., ammonium nitrate and potassium nitrate in MS medium compared to the other media could also have played an important role in the growth and development of protocorms. The importance of ammonium and nitrate ions (both individually and in combination) during seedling development has been well established (Arditti and Ernst 1984). Though nitrogen might not have had a significant role on seed germination of *C. eburneum* but its stimulatory effect on protocorm development and growth was observed in the present study. The presence of Fe-EDTA in the media as a growth promoter contributes to healthy growth of the protocorms into plantlets (Dutta et al. 2011). The suitability of MS medium for protocorm development into seedlings has also been reported by Shadang et al*.* (2007).

### Effect of growth regulators on seedling growth and development

Growth regulators have profound effect on seedling growth of orchids (Roy et al. 2011). Cytokinins enhance shoot regeneration and auxin induces root development in shoots to make it a complete plant. In the present study, it was found that the seedlings in MS medium supplemented with 10 μM BAP and 15 μM IAA singly showed the best response (Table 2; Fig. 1d, e). The beneficiary effect of BAP on the growth and development of orchids has been reported by several workers (Basker and Narmatha Bai 2010; Hossain et al. 2010; Roy et al. 2011)*.* In the present study, when the seedlings of *C. eburneum* were cultured in MS medium supplemented with TDZ, rooting was inhibited and at 20 μM TDZ in the medium, the seedlings died. However, Khampa et al*.* (2010) found that in *Grammatophyllum speciosum*, TDZ promoted protocorm formation. In the present study, the effect of Kn on seedling was similar to that of TDZ. These results are in agreement to those reported by Basker and Narmatha Bai (2010) on *Eria bambusifolia*. It was also observed that with further increase in the concentration of both cytokinins and auxins, growth of seedlings of *C. eburneum* was inhibited. Nagaraju et al. (2003) had also reported that cytokinins at higher concentrations have inhibitory effect on the growth of the plant. It was observed that when the medium was supplemented with cytokinins and auxins alone, the seedling’s response was poor but with auxins and cytokinins in combination, response of the seedlings was enhanced. When the seedlings were transferred to MS medium supplemented with BAP in combination with IAA and NAA, the growth and development of the seedlings were greatly enhanced at 15 μM each of BAP and NAA in the medium (Table 2). The synergistic effects of cytokinins and auxins in combination on orchids have been reported by many workers (Hossain et al. 2010; Roy et al. 2011).

**Table 2:** Effect of different growth regulators incorporated singly and in combination in MS medium on the growth and development of *C. eburneum* seedlings after 4 weeks of culture

Treatments (μM)						Average shoot number^#^	Average shoot length (cm)^#^	Average root number^#^	Average root length (cm)^#^
Control (MS)						2.1 ± 0.2^b^	2.0 ± 0.1^b^	1.5 ± 0.3^b^	2.3 ± 1.4^b^
*BAP*	*Kn*	*TDZ*	*NAA*	*IAA*	*2,4-* *d*				
5						3.2 ± 0.3^c^	3.9 ± 0.1^cd^	0	0
10						3.7 ± 0.2^c^	4.0 ± 0.1^d^	0.6 ± 0.3^a^	0.4 ± 0.2^a^
15						3.2 ± 0.2^c^	4.0 ± 0.1^d^	0.4 ± 0.3^a^	0.1 ± 0.1^a^
20						3.0 ± 0.3^bc^	3.0 ± 0.1^c^	0.1 ± 0.1^a^	0.1 ± 0.1^a^
	5					0.2 ± 0.1^a^	0.3 ± 0.2^a^	0.2 ± 0.1^a^	0.1 ± 0.1^a^
	10					0.4 ± 0.2^a^	0.8 ± 0.3^ab^	0.5 ± 0.3^a^	0.2 ± 0.1^a^
	15					0.7 ± 0.3^a^	0.9 ± 0.3^ab^	0.5 ± 0.3^a^	0.2 ± 0.1^a^
	20					0	0	0	0
	0	5				0.3 ± 0.2^a^	0.3 ± 0.2^a^	0	0
	0	10				0.5 ± 0.3^a^	0.4 ± 0.2^a^	0	0
	0	15				0.3 ± 0.2^a^	0.4 ± 0.2^a^	0	0
	0	20				0	0	0	0
			5			1.4 ± 0.2^ab^	2.9 ± 0.1^bc^	1.3 ± 0.2^ab^	1.4 ± 0.2^ab^
			10			2.0 ± 0.0^b^	2.8 ± 0.2^bc^	2.2 ± 0.2^b^	1.8 ± 0.2^ab^
			15			2.1 ± 0.1^b^	3.0 ± 0.2^c^	2.6 ± 0.2^b^	1.7 ± 0.1^ab^
			20			1.8 ± 0.2^b^	2.8 ± 0.1^bc^	1.4 ± 0.2^ab^	1.2 ± 0.2^a^
				5		2.2 ± 0.2^b^	1.9 ± 0.1^b^	0	0
				10		2.5 ± 0.3^b^	1.9 ± 0.2^b^	2.4 ± 0.4^b^	1.0 ± 0.2^a^
				15		2.7 ± 0.3^bc^	1.9 ± 0.2^b^	2.3 ± 0.4^b^	1.0 ± 0.2^a^
				20		2.2 ± 0.2^b^	1.7 ± 0.2^b^	0.4 ± 0.3^a^	0.2 ± 0.1^a^
					5	0	0	0	0
					10	0.1 ± 0.1^a^	0.1 ± 0.1^a^	0	0
					15	0.1 ± 0.1^a^	0.1 ± 0.1^a^	0	0
					20	0	0	0	0
5			5			2.1 ± 0.2^b^	1.5 ± 0.2^a^	0	0
5			10			2.8 ± 0.3^b^	2.0 ± 0.0^a^	0.3 ± 0.2^a^	1.6 ± 0.1^b^
5			15			2.6 ± 0.1^b^	2.0 ± 0.0^a^	1.3 ± 0.2^b^	1.6 ± 0.2^b^
5			20			2.8 ± 0.2^b^	2.0 ± 0.0^a^	1.0 ± 0.2^b^	1.3 ± 0.3^ab^
10			5			2.8 ± 0.2^bc^	2.8 ± 0.3^a^	1.9 ± 0.1^b^	2.0 ± 0.2^b^
10			10			3.0 ± 0.1^bc^	4.0 ± 0.0^b^	2.0 ± 0.0^bc^	1.8 ± 0.2^b^
10			15			3.7 ± 0.2^c^	4.0 ± 0.0^b^	2.4 ± 0.2^c^	2.4 ± 0.1^bc^
10			20			2.6 ± 0.1b^b^	4.0 ± 0.0^b^	1.9 ± 0.3^bc^	1.7 ± 0.2^b^
15			5			4.2 ± 0.3^cd^	4.1 ± 0.2^b^	2.2 ± 0.2^bc^	2.0 ± 0.3^b^
15			10			4.4 ± 0.1^cd^	5.5 ± 0.3^bc^	2.8 ± 0.3^bc^	2.2 ± 0.4^b^
15			15			5.2 ± 0.3^d^	6.0 ± 0.0^c^	4.0 ± 0.1^d^	3.0 ± 0.2^c^
15			20			3.2 ± 0.4^c^	4.0 ± 0.0^b^	3.4 ± 0.2^d^	2.5 ± 0.3^bc^
20			5			3.1 ± 0.3^bc^	2.4 ± 0.3^a^	1.5 ± 0.3^b^	0.7 ± 0.2^a^
20			10			2.2 ± 0.3^b^	2.0 ± 0.0^a^	1.2 ± 0.1^a^	0.5 ± 0.2^a^
20			15			0.7 ± 0.2^a^	2.0 ± 0.0^a^	0	0
20			20			0.4 ± 0.3^a^	1.7 ± 0.2^a^	0	0
5				5		2.0 ± 0.2^b^	1.3 ± 0.7^a^	0	0
5				10		2.2 ± 0.2^b^	1.6 ± 0.5^a^	0	0
5				15		2.3 ± 0.2^b^	1.8 ± 0.8^ab^	0	0
5				20		2.5 ± 0.2^b^	1.6 ± 0.5^a^	0	0
10				5		2.4 ± 0.2^b^	2.2 ± 0.8^b^	0	0
10				10		2.8 ± 0.1^bc^	3.2 ± 1.0^cd^	0.7 ± 0.2^a^	0.8 ± 0.2^a^
10				15		3.3 ± 0.1^c^	4.7 ± 0.9^d^	0.7 ± 0.3^a^	0.8 ± 0.3^a^
10				20		2.3 ± 0.1^b^	1.9 ± 0.3^ab^	0	0
15				5		2.5 ± 0.2^bc^	2.6 ± 0.3^b^	0.8 ± 0.1^a^	0.8 ± 0.2^a^
15				10		3.4 ± 0.2^c^	4.4 ± 0.3^d^	1.3 ± 0.2^ab^	1.6 ± 0.2^ab^
15				15		2.3 ± 0.1^b^	4.4 ± 0.3^d^	2.4 ± 0.2^b^	2.0 ± 0.2^ab^
15				20		2.4 ± 0.2^bc^	2.9 ± 0.3^cd^	1.4 ± 0.2^b^	1.5 ± 0.3^ab^
20				5		1.1 ± 0.2^a^	2.4 ± 0.3^b^	1.0 ± 0.0^ab^	1.1 ± 0.2^a^
20				10		1.7 ± 0.2^ab^	1.6 ± 0.2^a^	1.0 ± 0.0^ab^	1.1 ± 0.1^a^
20				15		0.8 ± 0.2^a^	1 ± 0^a^	0.4 ± 0.2^a^	0.4 ± 0.2^a^
20				20		0.5 ± 0.2^a^	1 ± 0^a^	0	0

Values are mean ± SEM of three experiments with ten replicates/experiment

ANOVA test shows that growth and development of seedlings is highly significant at 5 % level

Means followed by the same letter are not significantly different according to Tukey’s test (*p* = 0.05)

^#^Data shown are the mean of ten replicates ± standard error (SE)

### Acclimatization and transfer of in vitro-raised plantlets to soil

Complete plantlets when grown in the compost mixture comprising brick, charcoal and decaying litter in the ratio 1:1:1 and a layer of moss on top showed highest survivability of 70 % as compared to other compost mixtures used (Fig. 1f). This might be due to the fact that the roots were given structural support, and the presence of air spaces between the substratum particles facilitated the roots to spread out properly. Also, the required moisture content was maintained by the layer of moss on top (Nongrum et al. 2007). During acclimatization of the plantlets, it was observed that the original shoots senesced and a new shoot emerged from the corm upon transfer in the potting medium. Similar results have also been reported in orchids (Kauth et al. 2006; Dutra et al. 2008).

## Conclusion

From the present study, it may be concluded that the requirements of nutrients for complete seedling development through asymbiotic seed germination vary at different stages of growth and development. The protocol developed in the present study can be used for *ex vitro* conservation of *C. eburneum,* a threatened and vulnerable orchid species, through asymbiotic seed germination.
